# Facilitators and barriers to effective primary healthcare and family medicine in Namibia

**DOI:** 10.4102/phcfm.v17i1.5102

**Published:** 2025-11-17

**Authors:** Daniel O. Ashipala, Fransisco C. Ntjamba, Fillemon S. Albanus

**Affiliations:** 1 Department of General Nursing Sciences, Faculty of Health Sciences and Veterinary Medicine, University of Namibia, Windhoek, Namibia

**Keywords:** Namibia, community health services, delivery of health care, government, primary health care, social segregation

## Abstract

During independence in 1990, Namibia inherited a healthcare system that was deeply rooted in racial segregation and heavily centred on curative rather than preventive care. The delivery model was structured in two tiers: public healthcare under the Ministry of Health and Social Services (MoHSS) and private healthcare. Since the global recognition of primary health care (PHC) at the Alma-Ata conference in 1978, PHC has served as the foundation and the cornerstone of the global strategy for achieving ‘Health for All’. The MoHSS adopted this model at independence and has since relied on it to guide major health sector reforms. One of the government’s key objectives has been to correct historical imbalances by reallocating resources towards underserved regions. This has involved shifting the focus from hospital-based curative care to more preventive and community-based services delivered through local clinics, mobile health teams and community health volunteers.

## Overview of country and burden of disease

Primary health care (PHC) has been internationally recognised since the Alma-Ata (1978) and Astana (2018) Declarations as a comprehensive strategy to achieve ‘Health for All’. Primary health care represents an all-of-society approach to health, integrating principles of equity, participation, intersectoral collaboration and sustainability. Within this broad framework, primary care services are one essential component, delivered mainly through health facilities by nurses, doctors and allied health professionals. During independence in 1990, Namibia inherited a fragmented, racially segregated health system focused on curative hospital-based care.^1^ Since then, the government has adopted PHC as the foundation of health sector reform, emphasising preventive, community-oriented services.

Community-based health care (CBHC) operationalises PHC by providing services within communities. Community-based health care includes community health workers (CHWs), who connect households with the health system, provide health education, promote prevention and facilitate referrals to clinics and hospitals. The Ministry of Health and Social Services (MoHSS) leads service delivery; however, non-governmental organisations (NGOs), faith-based organisations (FBOs), community-based organisations (CBOs) and the private sector extend the reach of PHC, particularly in underserved areas. This highlights Namibia’s adoption of a comprehensive PHC model, rather than a selective one.

Namibia is geographically large (824 300 km^2^), but sparsely populated, with an estimated population of 3.09 million in 2025.^2^ Population density is about three people per square kilometre, with concentrations along the northern border regions.^1^ Children under 15 years represent 37% of the population, and life expectancy at birth is 64 years, with women living longer than men. The leading causes of premature mortality are human immunodeficiency virus (HIV) and/or acquired immunodeficiency syndrome (AIDS), neonatal conditions, respiratory illnesses, diarrhoea diseases, tuberculosis, road traffic injuries, ischaemic heart disease, strokes, violence-related injuries and diabetes.^3^

A surge in communicable diseases such as hepatitis E, cholera and coronavirus disease 2019 (COVID-19) has been accompanied by a growing burden of non-communicable diseases, and there is also an increasing prevalence of cancers, mental health disorders, substance abuse and suicide. Sexual and reproductive health issues also persist, although progress has been made through the implementation of the Prevention of Mother-to-Child Transmission (PMTCT) programme and the widespread availability of free antiretroviral therapy (ART) for all HIV-positive individuals.^4^ Communicable diseases such as HIV and/or AIDS and tuberculosis continue to strain the health sector,^5^ while malaria and tropical diseases also remain endemic in some regions.^6^

Facilitators of effective PHC in Namibia include strong government policy support, international partnerships and expanded health infrastructure,^5^ while barriers include a shortage of trained healthcare professionals, limited access to continuous professional development (CPD), inadequate medical resources, poor service integration and financial barriers.^7^

The MoHSS aspires to be the foremost provider of high-quality health and social services, aligning its operations with internationally recognised standards and global initiatives such as the Sustainable Development Goals (SDGs), which aim to improve human development by addressing key social determinants of health. Of particular relevance is SDG 3, which emphasises ensuring good health and well-being. The PHC workforce is largely nurse-led, especially in rural areas. In urban areas, services are complemented by midwives and clinical officers, with doctors offering oversight and specialist support; however, there is a shortage of trained healthcare professionals.^6^

Namibia exceeds the World Health Organization’s (WHO’s) recommended minimum threshold of 2.3 health workers per 1000 population, reporting a ratio of approximately 3 per 1000. However, this aggregate figure conceals deep-rooted disparities in the distribution and regulation of the health workforce. A significant imbalance exists between the public and private sectors, with only 38% of health professionals serving in the public sector, which is responsible for providing care to nearly 85% of the population. This misalignment places considerable strain on public health facilities, particularly in rural and peri-urban areas where human resource shortages are most acute. Furthermore, many physicians actively engaged in private practice remain on the public payroll, a situation made possible by the absence of a clear regulatory framework governing dual practice. This lack of oversight not only undermines accountability but also contributes to inefficiencies in resource allocation across the system. While the MoHSS has taken commendable steps – such as recruiting additional staff, upgrading infrastructure and expanding specialised services – these interventions may yield limited impact if the underlying issues of workforce distribution and regulatory enforcement remain unaddressed.

## Weaknesses

A significant weakness within Namibia’s health system lies in the fragmented and inefficient pharmaceutical supply chain, which undermines consistent access to essential medicines. Despite spending over Namibian dollar (NAD) 1 billion on imported medicinal and pharmaceutical products in the year leading up to August 2022 – with 10% of the MoHSS budget allocated to pharmaceuticals – critical supply challenges persist. These include poor procurement planning, inadequate storage capacity and, most notably, the absence of a centralised overview of clinical supply needs, which limits effective forecasting and distribution. This situation is further exacerbated by the fragmentation between the public and private sectors, where limited data integration and regulatory oversight – especially concerning the rapid growth of urban-based private clinics – have led to parallel systems that reinforce inequity. Additionally, public servants frequently turn to private care, exhausting their medical aid benefits prematurely and often facing out-of-pocket costs, a reflection of poor service integration and financial protection. Collectively, these gaps highlight systemic weaknesses in supply chain governance, regulation and equitable access, threatening the overall effectiveness and sustainability of the country’s PHC system.

Namibia’s PHC system continues to face significant financial and systemic challenges. Out-of-pocket spending remains a notable barrier, exposing vulnerable households to catastrophic health costs.^1,8^ In Namibia, the cost of primary care services vary, depending on the type of provider (see Table 1). Government-run facilities offer services that are generally free or charge nominal fees. Certain groups, such as pregnant women, children under five and individuals with specific chronic conditions, often receive services free of charge. However, small user fees may still apply for consultations or medications, especially at higher-level public hospitals. Faith-based or mission health facilities also provide primary care services, typically charging moderate to low fees. These services are largely paid out-of-pocket by patients, although some costs may be subsidised through donor funding or religious organisations. Private health services cater to 15% of the population, primarily those with medical insurance and urban access, while 85% of Namibians rely on underfunded public services.^7^ Health system financing remains fragmented, with domestic public sources accounting for only 46.9% of current health expenditure as of 2021, while external donors and private contributions make up the rest.^7^ Furthermore, primary care receives only 22% of funding, undermining the PHC-oriented approach.^7,6^ Primary care is funded through a combination of sources, with the government being the main contributor, followed by households, private medical aid schemes and donors. The MoHSS manages significant portions of these funds to implement the PHC approach, while NGOs, CBOs and international partners also contribute, particularly through specific public health programmes and initiatives like mobile clinics. Additionally, the rural–urban disparity in access to trained health workers and specialist services poses a persistent challenge.^8^ Since 2019, the country has reported insufficient key commodities such as contraceptives, paracetamol, and vaccines.

**TABLE 1:** Health facilities profile in Namibia.

Characteristics	*n*
**Hospital facilities**	
Central hospitals	1
Intermediate hospitals	4
District hospitals	34
Mission hospitals	7
Rural hospitals	36

**Total**	**82**
**Primary health facilities**	
Clinics	322
Polyclinics	1
Mission clinics	13
City council/municipal clinics	112
Rural health centres	56
Private clinics	80

**Total**	**584**

*Source:* Ministry of Health and Social Services (MoHSS). Namibia Master Health Facility List (MFL). 2024. Available from: https://mfl.mhss.gov.na; Improving Primary Health Care (PHC). Country Profile – Namibia: Organisation of Services. Primary Health Care Performance Initiative (PHCPI). 2024. Available from: https://www.improvingphc.org/namibia-organisation-services

Note: Data were obtained from the Ministry of Health and Social Services and Namibia Master Health Facility List.

## Strengths

Despite these limitations, Namibia has demonstrated a strong commitment to improving the quality of care within the PHC framework. One major strength is the development and implementation of the National Quality Policy and Strategy (NQPS) by the MoHSS in partnership with the WHO, aimed at ensuring consistent, high-quality care across all levels of the health system.^3^ The NQPS is improving quality by promoting standardised clinical guidelines, strengthening supervision systems and encouraging continuous quality improvement practices at the facility level. It has also helped integrate performance monitoring and patient safety into routine primary care services, leading to more consistent and accountable care delivery. Notably, recent quality improvements have been supported not by donor funding, but through the introduction of domestic health taxes, reflecting a strategic shift towards sustainable, nationally-driven health financing. These funds have enabled critical investments in infrastructure, health worker training and service delivery, particularly in HIV and/or AIDS, maternal health and child immunisation. Furthermore, selected hospitals are now pursuing quality accreditation, and the use of performance-based data is strengthening accountability and responsiveness across the public health system – contributing to improved health outcomes and system resilience.

## Current place of family medicine in the health system

Namibia established its School of Medicine in 2010, becoming one of the more recent countries in sub-Saharan Africa to locally train medical doctors. Family medicine was integrated into the undergraduate curriculum in 2016, following the appointment of a family physician as head of the newly established Department of Family and Community Medicine. Since then, the School has produced multiple cohorts of medical graduates, with increasing interest in postgraduate training in family medicine and primary care.

The University of Namibia (UNAM) currently offers a Postgraduate Diploma in Family Medicine and PHC, designed to serve as an additional qualification for general medical practitioners. However, this diploma does not confer specialist status. To be formally recognised as a family physician (specialist in family medicine), a practitioner must complete a Master’s degree in Family Medicine, which is currently available only outside Namibia.

Although most family physicians practising in Namibia have obtained their specialist qualifications abroad, particularly in South Africa, they are employed across various sectors, including the MoHSS, private practice and NGOs. The MoHSS has increasingly acknowledged the critical role of family physicians in enhancing the quality of PHC, and has included them in the district health system roadmap as part of its commitment to strengthening services.

## Conclusion

Namibia’s PHC system has transformed significantly since independence, shifting from a racially segregated, curative-based model to a more inclusive, preventive and community-oriented approach. It is supported by strong government policies, international partnerships and structured health programmes. Namibia has made significant strides in shifting from a curative, hospital-based model to a PHC-oriented system supported by community-based strategies and the gradual introduction of family medicine. Facilitators include policy commitment, donor support and infrastructure development, while barriers include workforce shortages, financing gaps and systemic inequities. The inclusion of family physicians within primary care teams presents a unique opportunity to enhance person-centred, comprehensive and continuous care. Strengthening PHC and family medicine together will be critical for Namibia to achieve equitable, high-quality healthcare for all.
